# Dose dense doxorubicin plus cyclophosphamide in a modified KEYNOTE522 regimen for triple negative breast cancer

**DOI:** 10.1038/s41523-024-00643-5

**Published:** 2024-06-04

**Authors:** Nicholas Mai, Sara Myers, Sherry Shen, Stephanie Downs-Canner, Mark Robson, Larry Norton, Yuan Chen, Tiffany Traina, Nour Abuhadra

**Affiliations:** 1 Department of Medicine, Memorial Sloan Kettering Cancer Center, New York, NY USA; 2 Department of Surgery, Memorial Sloan Kettering Cancer Center, New York, NY USA; 3 Department of Epidemiology and Biostatistics, Memorial Sloan Kettering Cancer Center, New York, NY USA

**Keywords:** Breast cancer, Cancer immunotherapy, Chemotherapy

## Abstract

The KEYNOTE-522 (KN522) regimen for neoadjuvant treatment of triple negative breast cancer (TNBC) utilized q3week dosing for doxorubicin plus cyclophosphamide (AC); however, dose-dense AC (ddAC) has demonstrated superior overall survival (OS) compared to q3week AC in anthracycline and taxane-based regimens. We performed a retrospective analysis assessing the use of ddAC in KN522 and the impact of sequencing ddAC before or after carboplatin/paclitaxel (CbT) plus pembrolizumab on multiple outcomes. 128 patients with TNBC were included. Overall pathologic complete response (pCR) rate of 56%. Sequencing of ddAC vs CbT first showed no difference in pCR rate (ddAC 55% vs. CbT 58%, *p* = 0.77). However, ddAC first compared to CbT first correlated with a significant increase in the incidence of overall treatment delays (ddAC 70% vs. CbT 51%, *p* = 0.03), with cytopenias most frequent (ddAC 59% vs. CbT 31%, *p* = 0.001). ddAC in a modified KN522 regimen is safe, tolerable, and effective. Efficacy is comparable regardless of chemotherapy sequencing, but ddAC first is significantly associated with higher rates of treatment delays and cytopenias.

## Introduction

Standard of care systemic treatment for stage II-III triple negative breast cancer (TNBC) consists of neoadjuvant chemoimmunotherapy based upon the results of KEYNOTE-522 (KN522)^1–3^. During the clinical trial, patients in the intervention arm were treated prior to surgery with four cycles of pembrolizumab every 3 weeks (q3week) simultaneously with carboplatin and paclitaxel (CbT) followed by four more cycles of q3week pembrolizumab with either doxorubicin + cyclophosphamide (AC) or epirubicin + cyclophosphamide. However, multiple other studies demonstrated that dose dense administration of doxorubicin + cyclophosphamide (ddAC), which condenses the dosing schedule to q2week and is administered concurrently with growth factor support, has been associated with better outcomes compared to q3week dosed AC in other contexts. Specifically, CALGB9741 directly compared q3week AC vs ddAC for the adjuvant treatment of node-positive breast cancer and found that dose-density was significantly associated with improved disease-free survival (DFS) and overall survival (OS) at 3 years^4^. A large follow-up meta-analysis done by the Early Breast Cancer Trialists’ Collaborative Group (EBCTCG) that included 37,298 women across 26 randomized trials investigating both neoadjuvant and adjuvant breast cancer treatment corroborated these results, showing that ddAC was associated with improved OS compared to q3week AC^5^. As such, using ddAC as part of neoadjuvant treatment for TNBC in a modified KN522 regimen is a common modification, even though its safety and efficacy has not been evaluated in a randomized, prospective study^6^. Further, alternative sequencing of AC in relation to CbT in the KN522 regimen is also a common modification, but its effects on efficacy and toxicity have not been reported.

**Fig. 1:**
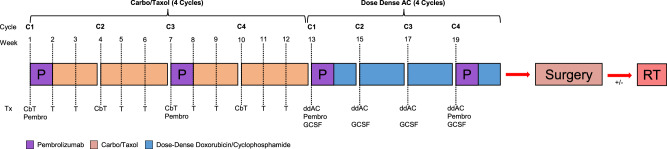
CbT first w/ ddAC w/ KN522 schema. A representative modified KN-522 regimen given at MSK outlined here. Pembrolizumab is given q6weeks at 400 mg flat dosing to best align with patient visits. Chemotherapy Dosing: CbT: weekly paclitaxel (80 mg/m2) and carboplatin (AUC 5) every 3 weeks; ddAC: Dose-dense doxorubicin (60 mg/m2) + cyclophosphamide (600 mg/m2) every 2 weeks with GCSF (granulocyte-colony stimulating factor), AKA peg-filgrastim.

Our institutional practice at Memorial Sloan Kettering Cancer Center (MSKCC) for the neoadjuvant treatment of TNBC involves a modified KN522 regimen where patients undergo four cycles of CbT q3week followed or preceded by (depending upon provider preference) four cycles of ddAC, all given concurrently with pembrolizumab 400 mg given q6weeks. This dose-schedule adjustment was designed to both provide dose-dense chemotherapy and to minimize the number of asynchronous visits for patients (Fig. 1). Here we report our institutional experience of patients treated at MSKCC with ddAC in this modified KN-522 regimen, and we also present a retrospective study of this regimen’s real-world feasibility, safety, and efficacy endpoints, including exploration of whether sequencing ddAC before or after CbT impacts efficacy and toxicity.

## Results

### Patients and treatment

From August 2021 to September 2022, 128 patients with TNBC met eligibility as defined above. Baseline demographics are outlined in Table 1. Median age at diagnosis was 50 (range 22–83 years). Within our cohort, the majority of patients were clinical stage II at diagnosis: Stage I 5%, Stage II 84%, and Stage III 11%. Of note, two patients had cT0N1 disease, both with biopsy-proven nodal TNBC with occult breast primary. While 6 patients technically had Stage I disease with cT1N0 tumors, all cases were treated as higher-stage disease due to case complexity, with almost all cases discussed at an institutional tumor board before proceeding with neoadjuvant KN522. Of the 6 patients, one patient had a disproportionately larger tumor (>2 cm) on palpation than noted on imaging (1.7 cm) and was treated as such. Another patient was started on KN522 given palpable axillary masses, but due to scheduling delays was unable to get an axillary biopsy until after starting chemotherapy, at which point the biopsy was negative for TNBC. One patient’s breast tumor was <2 cm but was metaplastic TNBC by histology and was started on KN522 based upon our institutional experience with metaplastic breast cancers in our Rare Breast Cancer Program^7^. The final three patients presented with multifocal or bilateral T1 tumors and were treated as higher risk disease.

**Table 1:** Demographics

Demographics:	All patients:	ddAC First:	CbT First:
Median Age, years (range)	50.2 (22.7–83.5)	51.7 (27.5–79.6)	49.6 (22.7–83.5)
Female Gender, n(%)	128 (100)	69 (100)	59 (100)
ECOG Performance Status, n(%)	122 (95.3)	65 (94.2)	57 (96.6)
Median BMI, kg/m2 (Range)	26 (17.1–45.8)	26.4 (18.1–39.4)	25.1 (17.1–45.8)
**Race or Ethnicity, n(%)**			
White	77 (60.2)	44 (63.8)	33 (55.9)
Asian	18 (14.1)	8 (11.6)	10 (16.9)
Black	23 (18.0)	11 (15.9)	12 (20.3)
Native American	0 (0)	0 (0)	0 (0)
Other	6 (4.7)	3 (4.3)	3 (5.1)
Refused to Answer	1 (0.8)	0 (0)	1 (1.7)
Unknown	3 (2.3)	3 (4.3)	0 (0)
Hispanic	9 (7.0)	6 (8.7)	3 (5.1)
**Baseline Labs:**			
Median Baseline Hgb (range)	12.8 (9.7–16.6)	13 (10.5–16.6)	12.8 (9.7–14.6)
Median Baseline Plt (range)	264 (116–505)	264 (116–505)	269 (167–431)
Median Baseline ANC (range)	4 (2–7.8)	264 (1.5–7.7)	3.8 (1.7–7.8)
**Clinical Staging, n(%)**			
Stage I	6 (4.7)	4 (5.8)	2 (3.4)
Stage II	108 (84.4)	58 (84.1)	50 (84.7)
Stage III	14 (10.9)	7 (10.1)	7 (11.9)
**T Stage, n(%)**			
T0	2 (1.6)	1 (1.4)	1 (1.7)
T1	27 (21.1)	14 (20.3)	13 (22.0)
T2	84 (65.6)	47 (68.1)	37 (62.7)
T3	10 (7.8)	3 (4.3)	7 (11.9)
T4	5 (3.9)	4 (5.8)	1 (1.7)
**N Stage, n(%)**			
N0	68 (53.1)	39 (56.5)	29 (49.2)
N1	55 (43.0)	28 (40.6)	27 (45.8)
N2	1 (0.8)	1 (1.4)	0 (0)
N3	4 (3.1)	1 (1.4)	3 (5.1)

All patient demographics are summarized here, both in aggregate and separated by chemotherapy sequence.

Almost all patients had an Eastern Cooperative Oncology Group performance status (ECOG PS) of 0 (122, 95%), while the remaining patients had PS of 1. The vast majority of patients (91) had no active comorbidities at time of diagnosis (71%), while 26 patients (20%) had at least one comorbidity and 11 patients (9%) had two comorbidities of those listed above. No patients had more than two concurrent active comorbidities at the time of treatment. Regarding germline mutation status, 18 patients (14%) had a germline *BRCA1* mutation, 1 patient (0.7%) had a germline *BRCA2* mutation, 24 patients did not have germline testing (19%), and the rest were *BRCA* negative. In total, 128 patients received ddAC and 1 patient received AC q3week during this time period at MSK (this patient was subsequently excluded from analysis). Of the 128 patients treated with ddAC, 54% received ddAC first while 46% received CbT first. Among all patients, 53.1% were treated with carboplatin q3week AUC 5, 40.6% were treated with carboplatin weekly AUC 1.5. Of the remaining patients, 5 (3.9%) started with a q3week schedule but had to switch to weekly due to toxicity, while 3 patients (2.3%) didn’t receive CbT at all due to toxicity from upfront ddAC. For all of the above treatment choices, chemotherapy sequence and choice of carboplatin regimen was decided by prescriber preference alone.

### Overall efficacy

**Fig. 2:**
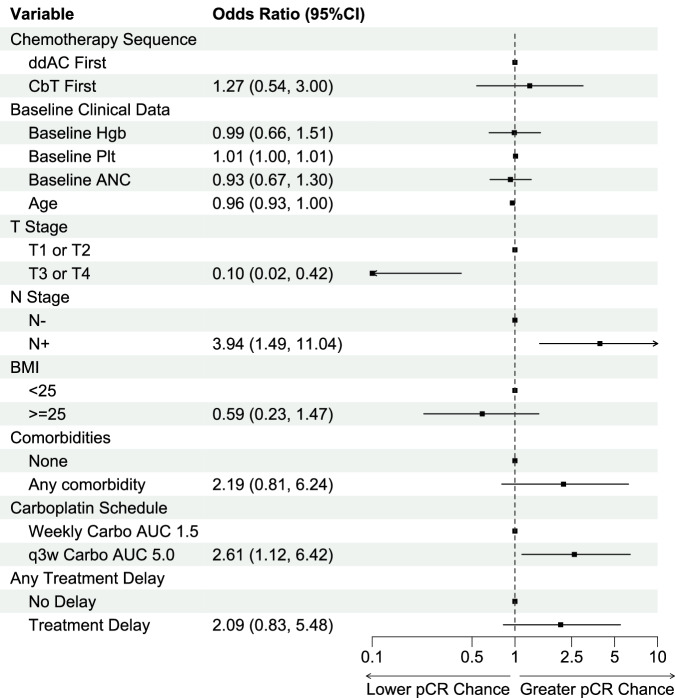
Clinical Variables Associated with pCR on Multivariate Analysis. Multivariable analysis showed that younger age, lower T stage, positive nodal status, and using q3week Carboplatin AUC 5.0 were significantly associated with higher chances of pCR. Notably, there was no difference in pCR based upon chemotherapy sequencing.

Overall pCR rate for all patients was 56%. Multivariable regression found younger age at diagnosis (*p* = 0.04), lower clinical T stage (*p* = 0.003), positive nodal status (*p* = 0.007), and q3week AUC 5 carboplatin dosing (*p* = 0.03) to be significantly associated with pCR in the overall cohort (Fig. 2). Of note, pCR rate was not affected by chemotherapy or immunotherapy treatment delays (No Delay 50% vs. Delay 60%, OR 2.07, 95%CI 0.80–5.52, *p* = 0.14), BMI (BMI ≤ 25 56% vs. BMI > 25 56%, OR 0.98, 95%CI 0.91–1.06, *p* = 0.7), or underlying comorbidities (no comorbidities 55% vs. comorbidities 59%, OR 2.05, 95%CI 0.76–5.84, *p* = 0.2).

### Overall toxicity and treatment delays

**Fig. 3:**
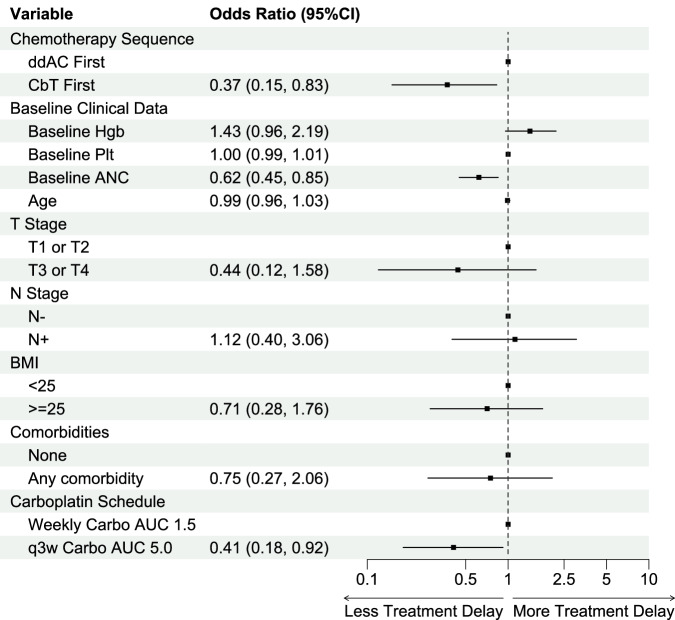
Clinical variables associated with treatment delays on multivariate analysis. Multivariate analysis showed that sequencing ddAC first, having a lower baseline ANC, and weekly carbo AUC 1.5 were all associated with higher incidence of treatment delay, with cytopenia (specifically neutropenia) being the most frequent cause of delay.

Treatment-related toxicity leading to chemotherapy delays occurred in 61% of total patients treated and immunotherapy delays occurred in 30%. Treatment related toxicity leading to any treatment component delay occurred in 70% of all patients. Of chemotherapy delays, the overwhelming majority were due to cytopenias (75%). Neutropenia, either by itself or presenting as bicytopenia or pancytopenia accounted for 86% of all cytopenias (Table 2). Of note, 4 patients experienced chemotherapy treatment delays due to immunotherapy toxicity alone (2 patients with hepatitis, 1 with dermatitis, 1 with adrenalitis). Of immunotherapy delays (38 patients, 30% of total), adrenal insufficiency (13% of IO delays) and hepatitis (18% of IO delays) were the most common immune-related toxicities leading to delays. 33 patients (26%) had adverse events (AEs) severe enough to require discontinuation of any treatment component, with carboplatin being the most commonly discontinued treatment component due to cytopenias. Of note, 3 patients (2.5%) who started with ddAC had AEs significant enough to either stop further dosing of ddAC or prohibit initiation of CbT and proceeded straight to surgery. Of the 33 patients who had treatment discontinuation, 6 patients (5%) had to stop pembrolizumab due to AEs. From multivariable analysis, a higher baseline ANC (*p* = 0.004) and using q3week carboplatin AUC 5 as opposed to weekly carboplatin AUC 1.5 (*p* = 0.03) were significantly associated with lower rates of treatment delays (Fig. 3).

**Table 2:** Chemotherapy Delay Breakdown by Cause

		Overall (*N* = 128)	ddAC First (*N* = 69)	CbT First (*N* = 59)
**Total Chemotherapy Delays,** ***n*(%)**		**48 (69.6)**	78 (60.9)	30 (50.9)
	Due to Chemotherapy Only, *n*(%)	62 (38.4)	42 (60.9)	20 (33.9)
	Due to IO Only, *n*(%)	4 (3.1)	1 (1.4)	3 (5.1)
	Due to Both, *n*(%)	9 (7.0)	4 (5.8)	5 (8.5)
	Due to Other, *n*(%)	3 (2.3)	1 (1.4)	2 (3.4)
**Chemotherapy-Related Adverse Event,** ***n*(%)**		46 (66.7)	71 (55.5)	25 (42.4)
	Cytopenia, *n*(%)	53 (41.4)	39 (56.5)	14 (23.7)
	Neutropenia, *n*(%)	33 (25.8)	24 (34.7)	9 (15.3)
	Anemia, *n*(%)	3 (2.3)	2 (2.9)	1 (1.7)
	Thrombocytopenia, *n*(%)	4 (3.1)	4 (5.8)	0 (0)
	Bicytopenia, *n*(%)	8 (6.2)	4 (5.8)	4 (6.8)
	Panctyopenia, *n*(%)	5 (3.9)	5 (7.2)	0 (0)
	Neutropenic Fever, *n*(%)	6 (4.6)	2 (2.9)	4 (6.8)
	Non-Neutropenic Fever, *n*(%)	1 (0.7)	1 (1.4)	0 (0)
	Neurologic, *n*(%)	5 (3.9)	1 (1.4)	4 (6.8)
	Gastrointestinal, *n*(%)	4 (3.1)	2 (2.9)	2 (3.4)
	Acute Kidney Injury, *n*(%)	1 (0.8)	0 (0)	1 (1.7)
	Hypersensitivity, *n*(%)	1 (0.8)	1(1.4)	0 (0)

A summary of chemotherapy delays and treatment toxicity leading to delays is arrayed here. While patients could have been affected by multiple toxicities simultaneously, documented here is the primary reason outlined for treatment delay or discontinuation per medical documentation. As a result, while “neutropenic fever” automatically assumes “neutropenia,” in this table they are considered separate events in the interest of clarity. Notably, all patients who were affected by bicytopenia or pancytopenia included neutropenia as one of their affected cell lines.

### Effect of sequencing ddAC vs CbT first on efficacy, toxicity, and treatment delays

**Fig. 4:**
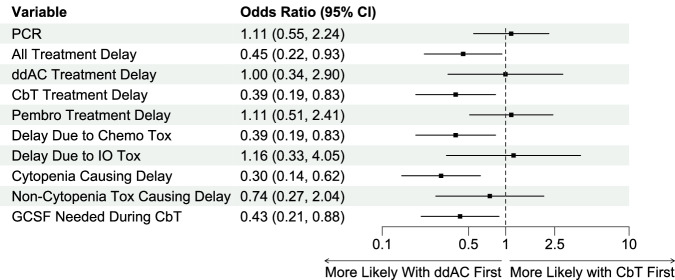
Univariate differences in efficacy and toxicity between sequencing ddAC vs. CbT first. When directly comparing the cohort of patients treated with CbT first to those treated with ddAC first, all treatment delays (and specifically cytopenias), delays during the CbT portion of KN522 (when given either first or second), delays attributed to chemotherapy toxicity, and the frequency of GCSF use during CbT were significantly associated with ddAC first. Notably, there was no difference in treatment delays during the ddAC portion of KN522 or pembrolizumab, delays due to immunotherapy related toxicity, or delays due to other medical reasons beyond cytopenias.

There was no difference in pCR rate between patients treated with ddAC or CbT first (ddAC 55% vs. CbT 58%, OR 1.25, 95%CI 0.55–2.89, *p* = 0.6). Regarding toxicity and treatment delays, the incidence of any chemotherapy delay at any point prior to surgery was significantly increased in patients treated with ddAC first compared to those treated with CbT first (ddAC 70% vs. CbT 51%, OR 0.37, 95%CI 0.15–0.83, *p* = 0.02), with treatment-limiting cytopenias being the most prevalent reason for treatment delay (83% of all delays). Among cytopenias, neutropenia either alone or presenting as bicytopenia or pancytopenia represented 88% of all cytopenias. Regarding specific components of the KN522 regimen, the incidence of CbT delays was 58% in patients treated with ddAC first and 27% in CbT first. Incidence of ddAC delays was 3% in patients treated with ddAC first and 7% in CbT first. Incidence of delays in both ddAC and CbT was 9% in ddAC first and 10% in CbT first (Fig. 4). There was no difference in the rate of immunotherapy delays (ddAC 26% vs. CbT 34%, OR 0.69, 95%CI 0.32–1.47, *p* = 0.33) based on sequence of chemotherapy.

## Discussion

Our data from this real-world cohort of patients supports the feasibility and tolerability of ddAC as an alternative for q3week AC in a modified KN522 regimen for the neoadjuvant treatment of TNBC. With the understanding that comparing real-world and on-trial data is imperfect, we report an overall real-world pCR rate of 56%, which is comparable in scale to pCR seen in a variety of neoadjuvant chemoimmunotherapy trials for TNBC: KEYNOTE-522 63%^1^, Impassion031 69%^8^, NeoTRIPaPD-1 48.6%^9^, ETCTN10013 55.6%^10^, GeparNUEVO 53.4%^11^, iSPY2 60%^12^. Due to the relative recency with which immune checkpoint inhibitors were approved for the neoadjuvant treatment of TNBC, other large real-world cohort reports are scarce, though a multicenter real-world analysis of 63 TNBC patients treated with neoadjuvant chemoimmunotherapy, published in early 2023, reported a pCR rate of 34.9%^13^. Broadly, pCR rate has been demonstrated as an early surrogate for recurrence rate, where patients who achieve pCR have been shown to have recurrence rates <10%, while patients who fail to achieve pCR can have recurrence rates reported up to 50%^14,15^. In summary, our data demonstrates that substituting ddAC instead of q3week AC does not negatively impact the efficacy of neoadjuvant chemoimmunotherapy.

Regarding toxicity, we ultimately chose treatment delay due to toxicity as our endpoint of choice because it was a functional endpoint that best approximated clinically relevant toxicity in a real-world setting where widespread heterogeneity in patient circumstances and provider preferences prevented standardized adverse event assessment. While our data did not establish a statistically significant correlation between treatment delays and treatment efficacy, treatment delays are still independently clinically important because significant treatment delays have been associated with increased long-term mortality, increased financial burden to both patients and the health system, and patient distress^16^. While we do report an overall chemotherapy delay rate of 61% in all our patients, the vast majority of our patients were still able to complete the full KN522 regimen prior to surgery. The KEYNOTE-522 trial did not report their total number and proportion of treatment delays, but it did report that 23.3% of their patients required discontinuation of any trial drug due to AE^1^. Comparably, our cohort demonstrated that 30% of patients required complete discontinuation of any treatment component due to AE.

Taken together, our efficacy and toxicity data suggests that the sequence of administration of the KN522 regimen matters. While the sequence of ddAC vs CbT first did not yield any significant differences in pCR rate, there was a significant increase in the incidence of treatment delays and chemotherapy related toxicity in patients treated with ddAC first. Hematologic toxicity, especially neutropenia, was most pronounced. When comparing sequencing, 70% of patients treated with ddAC first compared to 51% of those treated with CbT first experienced treatment delays, with a statistically significant odds ratio of 0.37, favoring CbT first to limit treatment delays when compared to ddAC first. For patients suffering treatment delays due to neutropenia, almost all patients had grade ≥ 3 neutropenia. Clinically significant neutropenia occurred in 51% of all patients treated with ddAC first, which accounted for an overwhelming 73% of all documented treatment delays in this cohort. Conversely, for patients treated with CbT first, clinically significant neutropenia occurred in only 29% of these patients and accounted for 57% of all treatment delays in this cohort. Again, in comparison to results from the KEYNOTE-522 trial, the 29% incidence of toxicity seen in our CbT first cohort was comparable to KEYNOTE-522’s reported incidence of grade ≥ 3 neutropenia of 34.6%^1^.

Given the stark difference in hematologic toxicity between the two cohorts, we hypothesize that the difference in these toxicity profiles based upon chemotherapy sequencing may stem from reliance upon GCSF to maintain a dose-dense schedule. It is well known that GCSF mobilizes hematopoietic stem cells from the bone marrow to the periphery, and as such it is a critical component of modern methods for stem cell collection in preparation for bone marrow transplant^17,18^. While this mobilization is necessary to prevent dangerous neutropenia during the administration of ddAC, we hypothesize that this mobilization to the periphery also therefore increases stem cell exposure to cytotoxic agents. For patients treated with CbT first, this stem cell exposure is minimized because patients usually have a prolonged chemotherapy-free period upon completion of the last 4 cycles of ddAC, as they prepare for surgery, post-surgical recovery, and potential radiation. For patients treated with ddAC first however, the hematopoietic stem cells get exposure to both the initial 8 weeks of ddAC as well as another 12 weeks of carboplatin/paclitaxel.

A separate unexpected result from our analysis was that there were statistically significant differences in both pCR rates and the incidence of treatment delays based upon a patient’s carboplatin dosing schedule. Specifically, our data suggests that patients treated with q3week carboplatin AUC 5 had significantly higher rates of pCR and lower incidence of treatment delays compared to weekly carboplatin AUC 1.5. In both instances, this is contrary to conventional thought. Regarding efficacy, the most recent National Comprehensive Cancer Network (NCCN) guidelines recommend both dosing schedules interchangeably, and a number of retrospective analyses have not demonstrated clear difference in pCR based upon carboplatin dosing schedules^19,20^. Further, the results from KEYNOTE-522 demonstrated a statistically significant difference in pCR rates between placebo and intervention arms in the weekly carboplatin subgroup but did not show statistical significance in the q3week carboplatin subgroup^1^. However, in KEYNOTE-522, this analysis was univariate, and the two subgroups were not directly compared to each other as they were in our analysis via multivariate regression. Regarding toxicity, in routine clinical practice q3week carboplatin typically has more side effects than weekly carboplatin and, as such, younger, fitter patients are inherently selected for this regimen. To address both issues, we do acknowledge that our data is retrospective and non-randomized, so selection bias in this instance may play a role. However, this has been mitigated by our multivariable modeling, and we did not see any appreciable differences in patient age or ECOG status based upon carboplatin schedule. Additionally, there was a near-equal proportion patients treated with q3week carboplatin between the ddAC first (43.5%) and CbT first (43.1%) groups. Further, our institutional practice regarding q3week carboplatin is not to administer GCSF prophylactically and only if needed, so GCSF use could not explain the lower toxicity incidence. To try to explain the effect seen, we hypothesized that the bolus carboplatin dosing when given q3week may yield differential efficacy for our patients because it is almost always given on the same day as the higher dose pembrolizumab 400 mg q6week that we favor to minimize dyssynchronous patient visits. While this itself cannot be proven from our study alone, results from the TONIC trial have suggested that the chemotherapeutic partner given with PD-1 blockade is critical towards fostering a favorably inflammatory tumor microenvironment (with platinum and anthracyclines being favored since they upregulate immune-related genes)^21^. Additionally, the single-arm NEOPACT trial also yielded a pCR rate of 58% with a q3week neoadjuvant docetaxel, carboplatin AUC 6, and pembrolizumab regimen for TNBC, also potentially suggesting some synergy between pembrolizumab and higher AUC q3week carboplatin dosing^22^.

The key limitation of this study is its retrospective, non-randomized nature, which prevents us from making definitive conclusions based upon our data. However, when comparing the two main subgroups of our analysis (ddAC vs CbT first), we found relatively comparable distributions in key demographic metrics, including age, ECOG status, and baseline comorbidities in both univariate and multivariate analysis. Further, our demographic data also indicates that our patient population was overall quite healthy, with no patient with an ECOG > 1 and no patient with more than one of the major pre-existing comorbidities outlined above. This itself may indicate selection bias by providers regarding which patients to put on KN522 or may speak to the healthiness of MSK’s patient population, which both may affect broader generalizability. Another aspect affecting generalizability is the fact that MSK is a large cancer center with significant institutional resources to support the administration of dose-dense chemotherapy and intervention on its potential complications, and this degree of support may not be available at lower volume centers.

Overall, our real-world data demonstrates that ddAC is a reasonable modification to the KN522 regimen that maintains efficacy and toxicity expected from our current standard of care. Dose-density is a common modification for KN522 in modern clinical practice despite lack of significant evidence in chemoimmunotherapy regimens, and our study helps fill this knowledge gap by demonstrating feasibility and tolerability. Further, our real-world data provides guidance on optimal administration of this modification, as it also suggests that when substituting in ddAC for q3week AC, sequencing it after CbT is likely to be overall more tolerable with lower incidence of hematologic toxicity. Further, utilizing a bolus q3week carboplatin dosing schedule during the CbT portion of the regimen may be associated with higher rates of pCR if the carboplatin is given concurrently with pembrolizumab. In summary, for a modified KN522 regimen incorporating dose-dense chemotherapy, we suggest starting with 4 cycles of q3weekly carboplatin AUC 5 and weekly paclitaxel given concurrently with q6week pembrolizumab 400 mg, followed by 4 cycles of dose-dense doxorubicin plus cyclophosphamide (Fig. 1) to both maximize chance of pCR and minimize hematologic toxicity.

## Methods

### Study population and patient selection

**Fig. 5:**
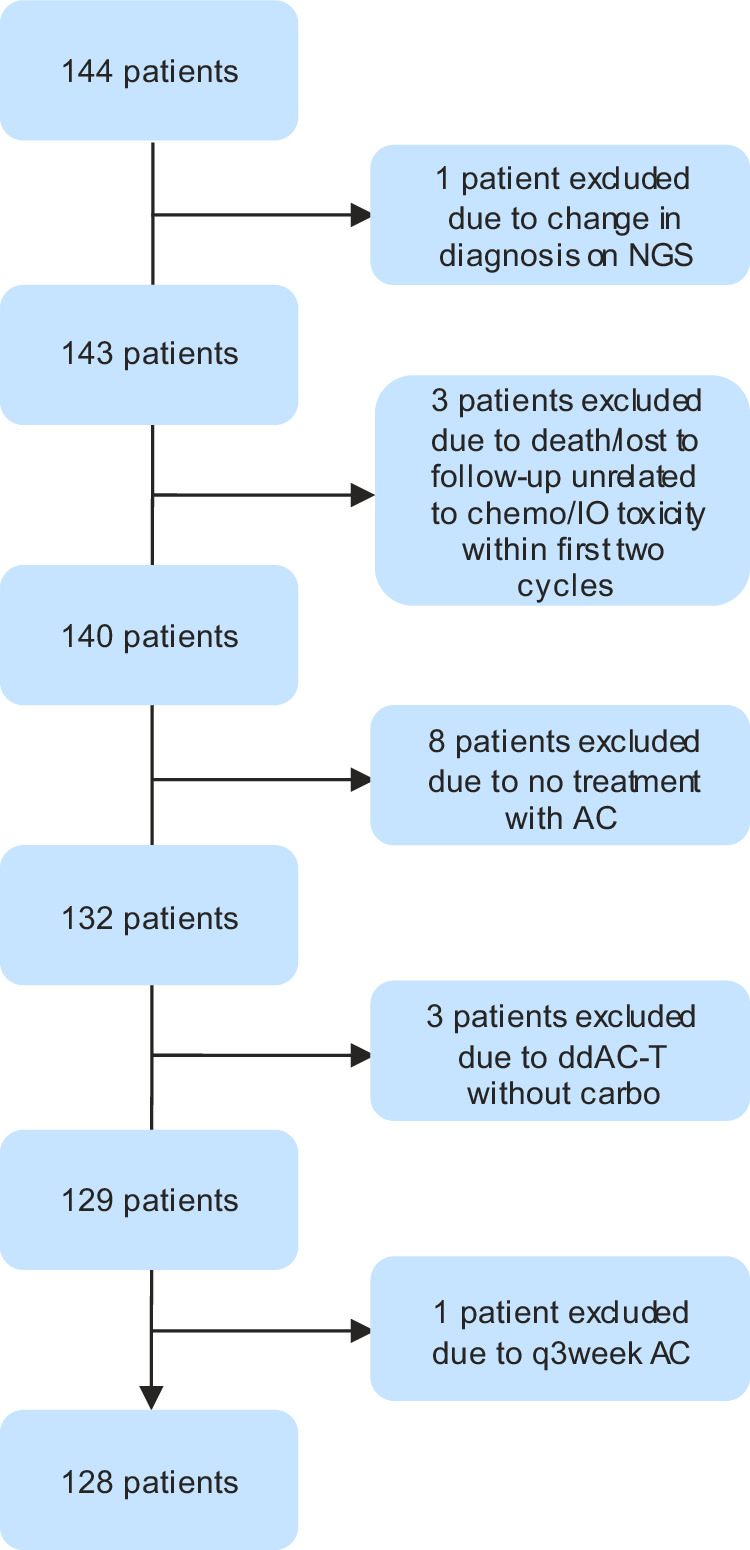
CONSORT diagram for patient exclusion from initial database query to final analysis group. The initial database included 144 patients with early-stage TNBC documented in the chart as being treated with neoadjuvant chemotherapy and immunotherapy. One patient was originally included in the analysis because she began ddAC for TNBC, but molecular analysis re-diagnosed her as diffuse large B-cell lymphoma and she was subsequently excluded from our analysis. Three further patients were excluded due to death or loss to follow-up unrelated to treatment toxicity within the first two cycles of therapy. Of the remaining 140 patients, since our primary research hypotheses revolved around ddAC in a modified KN-522 regimen, we included all patients that started treatment with the intention of completing the full course of ddAC, CbT, and pembrolizumab before surgery. As such, 2 patients who only received ddAC and could not receive TC due to toxicity were included in the analysis because they were planned for the full KN-522 at treatment start. On the contrary, 11 more patients were excluded due to exclusion of a major regimen component at treatment start (8 did not receive any AC due to prior anthracycline exposure or poor functional status, and 3 patients were planned for ddAC-T without carboplatin). We were left with 129 patients; 1 out of the remaining 129 patients was treated with q3week AC and was excluded to focus our analysis on ddAC with our remaining 128 patients.

Patients with TNBC treated at MSK between August 2021 and September 2022 with a preoperative KN522 regimen were eligible. Since this study specifically aimed to assess treatment related dose delays or discontinuations, patients were included in analysis even if they could not complete the full regimen for any reason, as long as the pre-treatment intent was to give four cycles of AC and four cycles of CbT (in any sequence) with concurrent pembrolizumab. Clinicopathological and demographic data were obtained from chart review. Other underlying health conditions were accounted for in aggregate, and patients were classified as either having no comorbidities, or having comorbidities if they had one or more of the following concomitant conditions at time of breast cancer diagnosis that required active management and treatment: chronic kidney disease, liver disease, prior malignancy, benign hematologic disorder (including venous thromboembolism), autoimmune disease, major cardiovascular disease, major pulmonary disease, or diabetes mellitus. The MSK Institutional Review Board on Human Research approved the study (IRB 22-291 A). This study was not associated with a clinical trial. Participants were not required to provide informed consent as only de-identified information was collected. Patient exclusion and database trimming are outlined in the CONSORT Flow Diagram depicted in Fig. 5. This study complied with all relevant ethical regulations regarding patient data, in line with ethical norms and standards in the Declaration of Helsinki.

### Outcomes of interest

The primary goal of this study was to describe pathologic complete response (pCR) rate, incidence of treatment-related toxicities resulting in treatment delays, and type of toxicity associated with ddAC in combination with CbT and pembrolizumab. For patients with clinically node-positive disease at diagnosis, they were considered to have achieved pCR only if they achieved both nodal and primary breast pCR. Treatment delays for toxicity were defined as a >1 week delay in treatment or discontinuation of a treatment regimen component due to a medical reason per medical documentation. We report these outcomes across the entire patient population, but we also specifically assessed both pCR and treatment delays based upon sequencing of ddAC before or after CbT to see if chemotherapy sequencing influenced outcomes.

### Statistical analysis

Data were evaluated descriptively as means, medians, and ranges. Baseline characteristics, incidence and type of delays, and treatment outcomes were compared between ddAC first and CbT first using two sample non-parametric tests. After univariate analysis, multivariable regression was performed with pCR rate and treatment delays as outcome variables. Statistical significance was defined as *p* < 0.05.

### Reporting summary

Further information on research design is available in the Nature Research Reporting Summary linked to this article.

### Supplementary information

Reporting Summary

## Data Availability

Data are available upon reasonable request at the discretion of the corresponding authors. Access to datasets used in this study should be requested directly from the corresponding authors and will involve data access request forms via Memorial Sloan Kettering Cancer Center. Subject to the institutional review boards’ ethical approval, unidentified data may be made available as a test subset. Data analysis methods have been described thoroughly in the Methods section so they can be independently replicated.
