# Supplementary material for: Dose dense doxorubicin plus cyclophosphamide in a modified KEYNOTE522 regimen for triple negative breast cancer
Source: NPJ Breast Cancer. 2024 Jun 4;10:39. doi: 10.1038/s41523-024-00643-5 (PMC11150442; doi:10.1038/s41523-024-00643-5)
Supplement: Supplementary file 1 — Reporting Summary [file 41523_2024_643_MOESM1_ESM.pdf]

nature portfolio

Corresponding author(s): Nour Abuhadra

Last updated by author(s): 3/4/2024

Reporting Summary

Nature Portfolio wishes to improve the reproducibility of the work that we publish. This form provides structure for consistency and transparency in reporting. For further information on Nature Portfolio policies, see our Editorial Policies and the Editorial Policy Checklist.

Statistics

For all statistical analyses, confirm that the following items are present in the figure legend, table legend, main text, or Methods section.

n/a	Confirmed	
☐	☒	The exact sample size (*n*) for each experimental group/condition, given as a discrete number and unit of measurement
☒	☐	A statement on whether measurements were taken from distinct samples or whether the same sample was measured repeatedly
☐	☒	The statistical test(s) used AND whether they are one- or two-sided<br>*Only common tests should be described solely by name; describe more complex techniques in the Methods section.*
☐	☒	A description of all covariates tested
☐	☒	A description of any assumptions or corrections, such as tests of normality and adjustment for multiple comparisons
☐	☒	A full description of the statistical parameters including central tendency (e.g. means) or other basic estimates (e.g. regression coefficient) AND variation (e.g. standard deviation) or associated estimates of uncertainty (e.g. confidence intervals)
☐	☒	For null hypothesis testing, the test statistic (e.g. *F*, *t*, *r*) with confidence intervals, effect sizes, degrees of freedom and *P* value noted<br>*Give P values as exact values whenever suitable.*
☒	☐	For Bayesian analysis, information on the choice of priors and Markov chain Monte Carlo settings
☒	☐	For hierarchical and complex designs, identification of the appropriate level for tests and full reporting of outcomes
☒	☐	Estimates of effect sizes (e.g. Cohen's *d*, Pearson's *r*), indicating how they were calculated

Our web collection on statistics for biologists contains articles on many of the points above.

Software and code

Policy information about availability of computer code

Data collection	Data was collected using retrospective chart review. No custom code was used.
Data analysis	Microsoft Excel for Microsofe 365 Version 2308 was used for data storage and basic data analysis<br>R version 4.3.2 was used for statistical analysis

For manuscripts utilizing custom algorithms or software that are central to the research but not yet described in published literature, software must be made available to editors and reviewers. We strongly encourage code deposition in a community repository (e.g. GitHub). See the Nature Portfolio guidelines for submitting code & software for further information.

Data

Policy information about availability of data

All manuscripts must include a data availability statement. This statement should provide the following information, where applicable:

- Accession codes, unique identifiers, or web links for publicly available datasets
- A description of any restrictions on data availability
- For clinical datasets or third party data, please ensure that the statement adheres to our policy

Data are available upon reasonable request at the discretion of the corresponding authors. Access to datasets used in this study should be requested directly from the corresponding authors and will involve data access request forms via Memorial Sloan Kettering Cancer Center. Subject to the institutional review boards' ethical

approval, unidentified data may be made available as a test subset. Data analysis methods have been described thoroughly in the Methods section so they can be independently replicated.

Research involving human participants, their data, or biological material

Policy information about studies with human participants or human data. See also policy information about sex, gender (identity/presentation), and sexual orientation and race, ethnicity and racism.

Reporting on sex and gender	Sex was considered for this analysis but our patient cohort was entirely female, which is common for a female-predominant disease like breast cancer.
Reporting on race, ethnicity, or other socially relevant groupings	Race or Ethnicity was determined by patient self report and included the following categories: White, Asian, Black, Native American, Hispanic, Other, Refused to Answer.<br>Race as a potential confounder was evaluated and adjusted for with multivariable modeling.
Population characteristics	*Describe the covariate-relevant population characteristics of the human research participants (e.g. age, genotypic information, past and current diagnosis and treatment categories). If you filled out the behavioural & social sciences study design questions and have nothing to add here, write "See above."*
Recruitment	n/a
Ethics oversight	MSK Institutional Review Board

Note that full information on the approval of the study protocol must also be provided in the manuscript.

Field-specific reporting

Please select the one below that is the best fit for your research. If you are not sure, read the appropriate sections before making your selection.

☒ Life sciences ☐ Behavioural & social sciences ☐ Ecological, evolutionary & environmental sciences

For a reference copy of the document with all sections, see nature.com/documents/nr-reporting-summary-flat.pdf

Life sciences study design

All studies must disclose on these points even when the disclosure is negative.

Sample size	Due to this being a retrospective study, sample size was determined by the number of appropriate patients identified.
Data exclusions	The initial database included 144 patients with early-stage TNBC documented in the chart as being treated with neoadjuvant chemotherapy and immunotherapy. One patient was originally included in the analysis because she began ddAC for TNBC, but molecular analysis re-diagnosed her as diffuse large B-cell lymphoma and she was subsequently excluded from our analysis. Three further patients were excluded due to death or loss to follow-up unrelated to treatment toxicity within the first two cycles of therapy. Of the remaining 140 patients, since our primary research hypotheses revolved around ddAC in a modified KN-522 regimen, we included all patients that started treatment with the intention of completing the full course of ddAC, CbT, and pembrolizumab before surgery. As such, 2 patients who only received ddAC and could not receive TC due to toxicity were included in the analysis because they were planned for the full KN-522 at treatment start. On the contrary, 11 more patients were excluded due to exclusion of a major regimen component at treatment start (8 did not receive any AC due to prior anthracycline exposure or poor functional status, and 3 patients were planned for ddAC-T without carboplatin). We were left with 129 patients; 1 out of the remaining 129 patients was treated with q3week AC and was excluded to focus our analysis on ddAC with our remaining 128 patients.
Replication	n/a: retrospective study
Randomization	n/a: retrospective study
Blinding	n/a: retrospective study

Reporting for specific materials, systems and methods

We require information from authors about some types of materials, experimental systems and methods used in many studies. Here, indicate whether each material, system or method listed is relevant to your study. If you are not sure if a list item applies to your research, read the appropriate section before selecting a response.

Materials & experimental systems

n/a	Involved in the study
☒	☐ Antibodies
☒	☐ Eukaryotic cell lines
☒	☐ Palaeontology and archaeology
☒	☐ Animals and other organisms
☒	☐ Clinical data
☒	☐ Dual use research of concern
☒	☐ Plants

Methods

n/a	Involved in the study
☒	☐ ChIP-seq
☒	☐ Flow cytometry
☒	☐ MRI-based neuroimaging

Plants

Seed stocks	*Report on the source of all seed stocks or other plant material used. If applicable, state the seed stock centre and catalogue number. If plant specimens were collected from the field, describe the collection location, date and sampling procedures.*
Novel plant genotypes	*Describe the methods by which all novel plant genotypes were produced. This includes those generated by transgenic approaches, gene editing, chemical/radiation-based mutagenesis and hybridization. For transgenic lines, describe the transformation method, the number of independent lines analyzed and the generation upon which experiments were performed. For gene-edited lines, describe the editor used, the endogenous sequence targeted for editing, the targeting guide RNA sequence (if applicable) and how the editor was applied.*
Authentication	*Describe any authentication procedures for each seed stock used or novel genotype generated. Describe any experiments used to assess the effect of a mutation and, where applicable, how potential secondary effects (e.g. second site T-DNA insertions, mosiacism, off-target gene editing) were examined.*
